# Clinical Effectiveness of Faecal Immunochemical Test in the Early Detection of Colorectal Cancer—An Umbrella Review

**DOI:** 10.3390/cancers14184391

**Published:** 2022-09-09

**Authors:** Jakub Świtalski, Tomasz Tatara, Katarzyna Wnuk, Wojciech Miazga, Dagmara Karauda, Adrian Matera, Magdalena Jabłońska, Sylwia Jopek, Urszula Religioni, Mariusz Gujski

**Affiliations:** 1 Department of Health Economics and Medical Law, Faculty of Health Sciences, Medical University of Warsaw, 01-445 Warsaw, Poland; 2 Department of Health Policy Programs, Department of Health Technology Assessment, Agency for Health Technology Assessment and Tariff System, 00-032 Warsaw, Poland; 3 Department of Public Health, Faculty of Health Sciences, Medical University of Warsaw, 02-091 Warsaw, Poland; 4 School of Public Health, Centre of Postgraduate Medical Education of Warsaw, Kleczewska 61/63, 01-826 Warsaw, Poland

**Keywords:** faecal immunochemical test, colorectal cancer, screening, sensitivity

## Abstract

**Simple Summary:**

Colorectal cancer (CRC) is a commonly occurring neoplasm causing significant decrease in quality of life as well as premature death. Due to this fact, screening is crucial, as the prognosis is dependent on disease advancement. There are several screening tests available for this neoplasm. One of the most promising and increasingly utilised is the FIT (faecal immunochemical test). The aim of our umbrella review was to analyse available data regarding the efficacy of the test. In this review, secondary studies concerning parameters such as specificity and sensitivity have been included. The results of this study confirm the high usefulness of the FIT in early CRC detection. The authors were able to conclude that FIT is an effective and possible to introduce screening test, which may be especially significant regarding systemic changes in countries where CRC screening had been conducted using different methods.

**Abstract:**

**Introduction:** The colorectal cancer prognosis depends on the stage of the neoplasm; therefore, its early detection plays an important role. The aim of the study is evaluation of the sensitivity, specificity, and clinical effectiveness of the faecal immunochemical test in the early colorectal cancer detection. **Methods:** The clinical analysis was based on the results of the studies included in a systematic review conducted in accordance with the Cochrane Collaboration guidelines. The following medical information sources were searched: Medline (via PubMed), Embase (via Ovid), The Cochrane Library. **Results:** From 241 citations, 13 studies were included in this review. All included studies had a low risk of bias. The faecal immunochemical test is highly specific in all analysed populations ranging from 85% to 97%. In most of the found studies, sensitivity is over 75%. The faecal immunochemical test screening also determines a reduction in death (10–59%) due to colorectal cancer. **Conclusions:** The faecal immunochemical test is an effective and cost-effective method of conducting population-wide colorectal cancer screening. It is an alternative or complementary to other screening tests, including colonoscopy.

## 1. Introduction

Globally, colorectal cancer (CRC) is the third most common cancer (with a total of nearly 1.9 million new cases in 2020). The number of new cases in 2020 divided by cancer site was as follows (age-standardised in parentheses, incidence rate per 100,000 people, years based on the 1966 Segi-Doll World standard population):

- Colon—600,896 in men (13.1), 547,619 in women (10.0);
- Rectum—443,358 in men (9.8), 288,852 in women (5.6) [1].

According to data from the Institute for Health Metrics and Evaluation, the disability-adjusted life years (DALYs)/100,000 for different age groups had been presented for colon and rectum cancer. The DALY is utilised to determine the condition of people’s health and represents the total years lost due to untimely death or disability due to injury or disease. In 2019, a significant increase in the DALY value was observed in men 60–64 years of age (1265.00/100,000). High DALY values are prevalent in all older age groups. A slight decrease of the DALY was observed in the 90–94 and 95+ age groups, but the value does not fall below 2454.24/100,000. In women, a significant increase in the DALY value was observed in the age group of 70–74 (1231.88/100,000). The gradual increase in DALYs by approximately 200 applies to each subsequent age group, ultimately reaching the level of 2356.17/100,000 among women over 95 years of age [2].

The colorectal cancer prognosis depends on the stage of the neoplasm; therefore, its early detection plays an important role. The main screening methods are the faecal immunochemical test (FIT), the guaiac faecal occult blood test (gFOBT), as well as endoscopic examinations, i.e., a colonoscopy or sigmoidoscopy [3]. Efforts should be made to promote patient participation in research, with screening by non-invasive testing being one of the possible incentives.

This article focuses on FIT and its role in the early detection of CRC. Several types of tests appear in the analysed publications, including:

- OC-Sensor—quantitative (test principle: latex agglutination, measured as optical change);
- OC-Light—qualitative (immunochromatographic);
- OC-Hemodia—quantitative (latex agglutination, measured as optical change) or qualitative (visual particle agglutination) [4].

**Aim:** The aim of the study is to evaluate the sensitivity, specificity, and clinical effectiveness of FIT in the early detection of CRC. Due to the large amount of secondary evidence analysing colorectal cancer detection, the authors decided to perform an umbrella review, which is currently the highest possible form of scientific evidence in medicine.

This study was conducted because in the public eye of many countries, the selection of the optimal CRC screening test had been discussed. In certain countries, Poland amongst them, shifting from screening methods based solely on colonoscopies to a FIT-based method has been considered.

## 2. Material and Method

The clinical analysis was based on the results of the studies included in the systematic review carried out according to the following protocol:

- defining the criteria for including studies for reviews;
- development/verification of a research report search strategy;
- searching for sources of medical information/updating of searching for sources of medical information;
- finding full texts of scientific reports that are potentially useful in clinical analysis;
- selection of studies based on inclusion criteria for the review;
- research results development;
- qualitative synthesis consisting of the analysis of the statistical and clinical relevance of the results of studies included in the analysis.

The process of searching for clinical trials was based on a detailed protocol developed prior to the start of the trial. The systematic review was carried out according to the guidelines of the Cochrane Collaboration [5]. The criteria for including studies for review, the search strategy, the method of selecting the studies, and the planned methodology for carrying out the analysis were taken into account.

The analysis included clinical trials that met the criteria:

- population: general adult population;
- interventions: FIT;
- alternative technologies (comparators): unlimited;
- methodologies: meta-analyzes of randomised and/or observational trials; systematic reviews of randomised and/or observational studies;
- endpoints: to assess the sensitivity, specificity, and clinical efficacy of FIT.

The following databases were searched in the research process: Medline (by PubMed), Embase (by Ovid), and The Cochrane Library. The last search of the database was carried out on 28 February 2022 (Supplementary Material Table S1).

**Figure 1:**
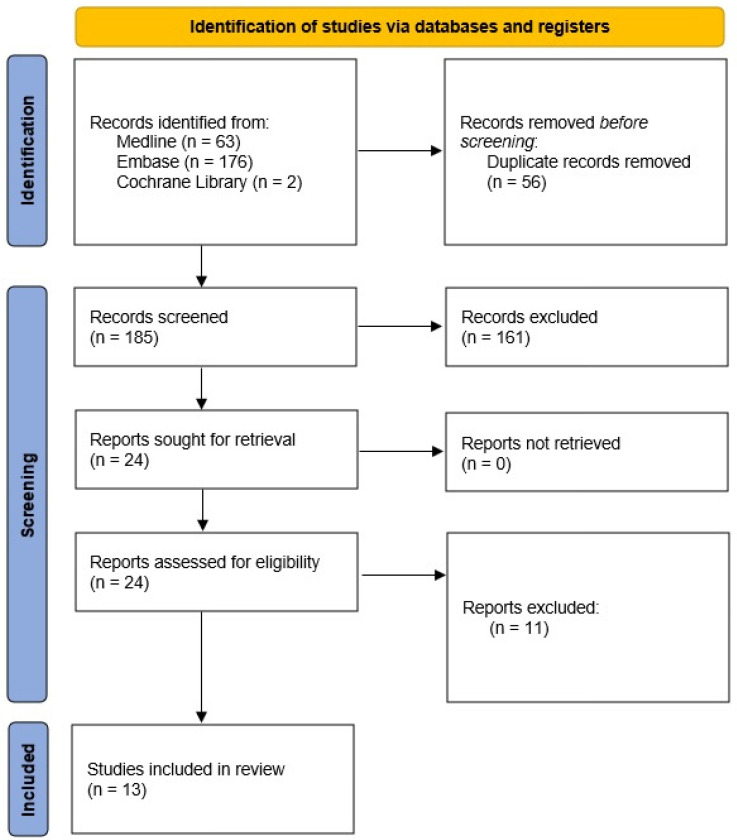
PRISMA flow diagram.

Research at all stages of the systematic review was selected by two independently working analysts (K.W. and A.M.). Any inconsistencies were resolved by consensus involving a third independent analyst (W.M.). The most common reasons for excluding studies from the analysis were related to the intervention (lack of a detailed FIT analysis) and methodology (lack of correct description of the material and method, incorrect synthesis of the review results). The study selection steps are shown in Figure 1. A list of included and excluded publications has been included in the Supplementary Material.

The included studies were assessed for quality and risk of error by validating the key domains of the AMSTAR2 systematic review tool [6]. The utilised tool allows for identification of the highest quality publications. To obtain the highest rating, the publication must positively conform to every statement. One negative score in the critical domain results in the systematic review rating being downgraded to “low”. Furthermore, two or more negative scores lower the evaluation of the study to “critically low”. The quality assessment was performed by two independently working analysts (M.J. and A.M.). Any inconsistencies were resolved by consensus involving a third independent analyst (J.Ś.). All included studies had a low risk of bias. Detailed results of the quality analysis and risk of error can be found in the supplementary material.

Secondary studies include the results of statistical analyses of individual studies. However, due to the fact that they are based on primary data, they are a reliable source of information. The results of each publication are presented individually.

## 3. Results

The criteria for inclusion in a systematic review, including the assessment of the sensitivity, specificity, and/or clinical effectiveness of FIT in the detection of CRC, met the following scientific evidence (n = 13; Forbes 2021, Lin 2021, Mutneja 2021a, Mutneja 2021b, Gini 2020, Meklin 2020, Niedermaier 2020, Zhong 2020, Imperiale 2019, Selby 2019, Stonestreet 2019, Katsoula 2017, Zhang 2017):

- Forbes 2021—a systematic review based on 8 observational studies, which analysed the impact of specific time intervals from a positive FIT result to colonoscopy on the presence of CRC, the presence of advanced CRC at diagnosis, overall mortality, and CRC mortality [7];
- Lin 2021—a meta-analysis of 223 publications (RCT and observational studies), which analysed the effectiveness and diagnostic precision of tests, and harms related to CRC screening as part of the USPSTF recommendation [8];
- Mutneja 2021a—5 RCT meta-analyses comparing the effectiveness of FIT with sigmoidoscopy in screening for CRC [9];
- Mutneja 2021b—a meta-analysis of 6 observational studies, evaluating the influence of the time after a positive colonoscopy following faecal occult blood test on CRC detection [10];
- Gini 2020—a systematic review of 18 RCTs and observational studies comparing the impact of CRC screening on mortality in European regions [11];
- Meklin 2020—a meta-analysis of 31 single-arm clinical trials assessing the diagnostic precision of FIT and gFOBT in screening tests [12];
- Niedermaier 2020—a meta-analysis of 44 observational studies, determining the diagnostic precision of FIT depending on the stage of CRC stage [13];
- Zhong 2020—6 RCT meta-analyses comparing the effectiveness of FIT and colonoscopy in detecting CRC in the intermediate-risk population [14];
- Imperiale 2019—a meta-analysis of 31 observational studies, defining the diagnostic precision of FIT in the detection of CRC and advanced colorectal adenomas in people from the intermediate risk group undergoing screening colonoscopy [15];
- Selby 2019—a meta-analysis of 46 observational studies, defining the diagnostic precision of FIT in the detection of CRC and advanced colorectal adenoma at different diagnostic thresholds in regards to gender and age [16];
- Stonestreet 2019—a meta-analysis of 17 observational studies, assessing the diagnostic precision of FIT in the detection of CRC in symptomatic and asymptomatic people [17];
- Katsoula 2017—a meta-analysis of 1 RCT and 11 observational studies, determining the diagnostic precision of FIT in the detection of CRC or advanced neoplasia of the large intestine in asymptomatic people at high risk [18];
- Zhang 2017—a meta-analysis of 44 RCTs and observational studies, evaluating the effectiveness of screening methods in preventing CRC disease and death [19];

The results of the included studies are presented below.

### 3.1. Sensitivity and Specificity of FIT in the Detection of CRC

The authors of the Lin 2021 meta-analysis determined the diagnostic precision of FIT depending on the type of test used. The observed observational studies described the results of the screening conducted in the population of asymptomatic people aged ≥ 40 years at the general risk of developing CRC. According to the results of the 13 publications meta-analysis, the sensitivity of the OC-Sensor test in terms of CRC detection is 0.74 [95% CI: (0.64; 0.83)], and the specificity is 0.94 [95% CI: (0.93; 0.96)]. However, the precision of the OC-Light test was determined on the basis of a meta-analysis of the results of 4 publications and amounted to 0.81 [95% CI: (0.70; 0.91)] for sensitivity and 0.93 [95% CI: (0.91; 0.96)] for specificity.

In the Meklin 2020 meta-analysis, the FIT diagnostic precision for the detection of CRC in the general population was determined based on the synthesis of the results of 24 publications. According to the results of the meta-analysis, the sensitivity of the FIT is 0.86 [95% Cl: (0.78; 0.93)] and the specificity is 0.85 [95% Cl: (0.81; 0.88)].

As part of the Niedermaier 2020 publication, the authors conducted a meta-analysis of 44 observational studies that determined the precision of FIT among people post-colonoscopy participants. Said study presents the results of the sensitivity and specificity of FIT tests depending on the studied population and the CRC stage determined during the diagnostic colonoscopy. According to the meta-analysis results, the overall sensitivity of the test in the detection of particular CRC advancement stages is 0.73 [95% Cl: (0.65; 0.79)] (stage I/A), 0.80 [95% Cl: (0.74; 0.84)] (stage II/B), 0.82 [95% Cl: (0.77; 0.87)] (stage III/C), 0.79 [95% Cl: (0.70; 0.86)] (stage IV/D). The specificity of the study for all patients who underwent colonoscopy was 0.89 [95% Cl: (0.85; 0.92)].

As part of the Imperiale 2019 meta-analysis, based on 31 observational studies, the diagnostic precision of FIT in the detection of CRC was determined for various values of the diagnostic threshold. It has been shown that, with a diagnostic threshold of <10 µg/g, the sensitivity is 0.78 [95% CI: (0.63, 0.88)] and the specificity is 0.90 [95% CI: (0.81, 0.95)]. At the diagnostic threshold of 10 µg/g, the sensitivity was 0.91 [95% CI: (0.84, 0.95)] and a specificity of 0.90 [95% CI: (0.86, 0.93)]. A diagnostic threshold of >10–<20 µg/g showed a sensitivity of 0.82 [95% CI: (0.63; 0.92)] and a specificity of 0.93 [95% CI: (0.91; 0.95)]. On the other hand, at the diagnostic threshold of 20 µg/g, the sensitivity was 0.75 [95% CI: (0.61; 0.86)], and the specificity was 0.95 [95% CI: (0.92; 0.96)]. With a diagnostic threshold of >20 µg/g, a sensitivity of 0.71 [95% CI: (0.56; 0.83)] and a specificity of 0.95 [95% CI: (0.94; 0.96)].

Selby’s meta-analysis, based on 46 observational studies, determined the diagnostic precision of the FIT test in the detection of CRC at various diagnostic thresholds in terms of gender and age. According to the results of the meta-analysis, the sensitivity of the FIT test is 0.76 [95% Cl: (0.72; 0.80)], and the specificity is 0.94 [95% Cl: (0.92; 0.95)]. In three observational studies where assessed the gender impact, the sensitivity of CRC detection was 0.77 [95% CI, (0.75; 0.79)] in men and 0.81 [(95% CI, (0.60; 100)] in women. The specificity reached 0.92 [95% Cl: (0.89; 0.95)] in men and 0.94 [95% Cl: (0.91; 0.97)] in women. According to the results of three observational studies with two age groups, the sensitivity of CRC detection for the age of 50–59 years was 0.85 [95% CI, (0.71; 0.99)], and for the age of 60–69 years was 0.73 [ 95% CI, (0.71; 0.75)]. Specificity, in turn, was estimated at 0.94 [95% Cl: (0.92; 0.97)] in the 50–59 age group and 0.93 [95% Cl: (0.90; 0.96)] in the 60–69 years group.

As part of the meta-analysis of Stonestreet 2019, based on 17 observational studies, the diagnostic precision of FIT in the detection of CRC was determined, showing the sensitivity and specificity at the level of 0.69 [95% CI: (0.54; 0.81)] and 0.94 [95% CI: (0.94; 0.95), respectively)]. On the other hand, the results of the 2017 Katsoul meta-analysis, including 1 RCT and 11 observational studies, showed a sensitivity and specificity of 0.93 [95% CI: (0.53; 0.99)] and 0.91 [95% CI: (0.59; 0.99)], respectively.

The following are the characteristics and results of studies on the diagnostic precision of FIT in the detection of CRC (Table 1).

### 3.2. Reporting for Screening and CRC-Related Detection, Occurrence, and Deaths

As part of the Mutneja 2021 meta-analysis based on 5 RCTs, the effectiveness of FIT was compared with sigmoidoscopy in screening conditions for CRC. It was shown that participation in the screening test using FIT was statistically significantly higher compared to the screening test using FS (*Flexible Sigmoidoscopy*) (OR = 2.11 [95% Cl: (1.29; 3.44)]). The publication presents different results in terms of the effectiveness of FIT compared to FS in the detection of CRC. The per-protocol analysis showed a statistically significant reduction in the CRC detection rate for FIT compared to FS-OR = 0.76 [95% CI: (0.61; 0.96)]. Based on the intention-to-treat (ITT) analysis, no statistical significance was demonstrated—OR = 1.15 [95% CI: (0.65; 2.02)]. The ITT analysis breaks down end-points into groups, to which test subjects had been randomly assigned regardless of whether subjects were ultimately subjected to planned intervention or not. This method allows keeping the idea of randomisation—starting balance of known and unknown prognostic variables between groups. 

Similar results were presented in the Zhong 2020 publication (meta-analysis of 6 RCT), which compared the effectiveness of FIT and colonoscopy in detecting CRC in the moderate-risk population. Participation in the FIT screening study was shown to be statistically significantly higher compared to the colonoscopy screening RR = 1.73 [95% CI: (1.29; 2.34)]. In the per-protocol analysis, a statistically significant reduction in the CRC detection rate of FIT-RR = 0.53 [95% CI: (0.33; 0.83)] was demonstrated. However, in the context of the ITT analysis, no statistical significance was shown—RR = 0.73 [95% CI: (0.37; 1.42)].

The characteristics and individual test results concerning the participation rates in the screening and the CRC detection rates are presented in Table 2.

In a systematic search conducted in Lin 2021, the authors also identified one cohort study assessing the effect of FIT screening on the risk of CRC death. According to the results, the FIT screening intervention method during the six-year follow-up period, compared to the lack of screening tests, statistically significantly reduces the risk of death by 10%—RR = 0.90 [95% Cl: (0.84; 0.95)].

In the Gini 2020 systematic review, it was shown that among those invited for FIT screening, CRC mortality was 36% lower compared to uninvited subjects. In turn, the probability of death due to CRC was 41% lower in the group participating in the FIT screening compared to those who did not participate. It was shown that the probability of death due to CRC in the FS study in combination with FIT was 25% lower in the invited group compared to the uninvited group—RR = 0.75 [95% Cl: (0.57; 0.99)].

In the Zhang 2017 meta-analysis, it was shown that screening with the use of the FIT test determines a statistically significant reduction in the incidence of CRC by 21% (RR = 0.79 [95% CI: (0.69; 0.92)]) and death due to CRC by 59% (RR = 0.41 [95 % CI: (0.29; 0.59)]).

The characteristics and results of studies on CRC deaths are presented in Table 3.

### 3.3. Time of Colonoscopy Measured from Positive FIT Result

The aim of Mutneja’s 2021b meta-analysis was to assess the impact of time passing from receiving a positive result of stool test to performing a diagnostic colonoscopy on the final detection of CRC. The publication authors analysed the results of 6 retrospective cohort studies (N = 361,637; 16,721 people with CRC and 3617 people with advanced CRC) comparing the CRC detection rates obtained later than a given cut-off point to the results obtained sooner than a given cut-off point (e.g., >1 month vs. ≤1 month). According to the results of the meta-analysis, no statistically significant differences were found in the 1-, 2-, and 3-month intervals between FOBT and colonoscopies in the detection of both CRC and advanced CRC. In regard to colonoscopies performed >6 months from receiving a positive FIT result, compared to colonoscopies <6 months, a statistically significant increased chance of detecting both CRC (OR = 1.58 [95% Cl: (1.23; 2.03)]) and advanced CRC (OR = 2.16 [95% Cl: (1.47; 3.16)]).

The Forbes 2021 publication is a systematic review of primary research also included in the Mutneja 2021b publication. The authors, despite the failure to perform a quantitative synthesis of the results, formulated a joint conclusion that a colonoscopy should not be performed later than 9 months after obtaining a positive FIT result. The authors also noted the additional time required to obtain a referral and surgical planning in the event of CRC detection and therefore proposed the best period for colonoscopy as shorter than 9 months after a positive FIT.

The studies included in the Forbes 2021 and Mutneja 2021b reviews included the analysis of CRC detection rates in the case of using the FIT OC-Sensor at a diagnostic threshold of 20 µg/g.

## 4. Discussion

Based on the results of the studies found in the systematic review, the sensitivity, specificity, and clinical effectiveness of FIT in the early detection of CRC were assessed.

The test is highly specific in all analysed populations (both symptomatic and asymptomatic), ranging from 85% to 97%. In most of the found studies, the sensitivity of FIT is over 75% [8,12,13,15,16,17,18]. The results of one of the studies indicate a reduction in the risk of death due to CRC as a result of screening with the use of FIT, compared to the lack of screening tests [8].

For discussion purposes, the current clinical practice guidelines for CRC screening were reviewed. The most important conclusions from the recommendations regarding screening with FIT are presented below.

The authors of the recommendations found agree that the main method of preventing the consequences of CRC is to perform screening tests aimed at early detection. Most clinical practice guidelines also note the significant role of FIT in the detection of CRC. Some organisations consider the FIT test to be one of the basic screening strategy, while other consider utilising the FIT test as an option depending on the clinical situation, usually at one or two-year intervals (ASCO 2019 [20], NHMRC 2017 [21], AAFP 2021 [22], ACG 2021 [23], ACS 2020 [24], USPSTF 2021 [25], ACP 2019 [26], ASGE 2017 [27], USMSTF2017a [28], CTFPHC 2016 [29]).

At the same time, several of the found guidelines emphasise that the best CRC screening method is colonoscopy due to its highest clinical effectiveness and the simultaneous possibility of removing polyps that can potentially lead to cancer development (ACG 2021, NCCN 2021 [30], ACS 2020, ACPGBI 2017 [31], ASGE 2017, USMSTF 2017a). Some organisations, highlighting the risk of potential harms related to colonoscopy tests, indicate the FIT and gFOBT tests as the preferred screening tests in the asymptomatic population (ASCO 2019, CCA 2018a [32], CCA 2018b [33], RACGP 2018 [34], UK NSC 2018 [35], USMSTF 2017b [36], NHMRC 2017, CTFPHC 2016, BCG 2016 [37]).

Despite the greater clinical effectiveness of colonoscopy in the detection of CRC, in most European Union countries, the first screening procedure used is stool examination—the FIT or gFOBT [38,39,40]. This is likely due to several factors, one of them being the test reporting level. The studies showed that people were more likely to participate in the FIT screening than in the screening using colonoscopy [14,41,42] or sigmoidoscopy [9]. In countries where CRC screening is based on FIT, the median reporting rate is 54% [95% CI: (49.28%; 58.69%)] [43]. For comparison in Poland, where the screening program is based solely on colonoscopy (in a system where the potential participant receives a personal invitation to the study), it is below 20% [44]. The lack of necessity to perform an endoscopic examination in the population of asymptomatic people causes an increase in the number of people willing to participate in screening. Moreover, it should be noted that colonoscopy requires the involvement of experienced medical personnel and the patient’s appearance in person at the facility performing the examination. Availability, simplicity, and the ability to self-collect samples for the FIT test can play an important role in maintaining high screening reporting rates. Another reason is the higher cost-effectiveness of the FIT compared to other screening tests. The results of cited studies indicate that FIT performed at two-year intervals is a cost-effective strategy [45,46,47].

It should be stressed that there are articles comparing the efficacy of FIT and gFOBT methods. In the secondary study by Lin 2021, it has been “in pooled values, commonly evaluated FITs and stool DNA with FIT performed better than high-sensitivity gFOBT to detect cancers” [8]. Few other studies also pointed to the advantage of the FIT method when compared to gFOBT [48,49,50,51]. The USMSTF 2017b recommendation underlines the specificity of the FIT in comparison to gFOBT in CRC and advanced neoplasm of the large intestine detection. Therefore, the organisation recommends choosing the FIT method over gFOBT [36]. The analysis of the European Colorectal Cancer Screening Guidelines Working Group guidelines currently points to FIT being the test of choice for population screening [52].

It should also be noted that conducting screening for CRC is a complex process in which many aspects must be taken into consideration. Cancer detection can be influenced by such factors as progression at the detection and performance of the FIT screening test based on the location of the tumour.

The efficacy of the screening tests is related also to the amount of interval CRCs. The interval CRCs percentage in the screening programmes utilising FIT had been estimated to be 7–23% amongst all detected CRC cases with a cut-off ranging from 10 to 20 µg/g depending on the study [53,54,55,56,57,58,59]. One of the most recent studies conducted in Belgium showed that interval CRCs after FIT usage were more common in women, in older age groups, in right-sided locations, as well as in the advanced stages of cancer [53]. In screening programmes utilising FIT, patients should be informed to conduct self-observation of any worrisome symptoms, even after a recent examination [60].

Additionally, using the FIT method is potentially related to surgical intervention possibilities. According to results from Italy, after implementing FIT-based screening programmes, the surgery rates decreased significantly (colonoscopy had been performed after a positive FIT result). During the first five years of the screening, the distal CRC resections insignificantly increased, and after that period, they significantly dropped by 9.1% annually. Similarly, in proximal colon cancer, in the beginning, an increase (by 5.8%) of the factor had been noted, and after a five-year period, a 4.1% drop had been observed [61,62] Similar results had been observed in other studies conducted in Italy [63]. The discernible difference between regions was based on the conducting of screening programmes (in regions without screening programmes, the surgery rates were higher) [61,62]. A different study, where surgery rates in patients with nonmalignant cancers had been analysed, showed the small value of the factor, and the surgical interventions had been performed mainly on the unresectable polyps [64].

## 5. Review Limitations

Only publications in English were included in the review. The search has been limited to publications in the last 10 years (28 February 2012–28 February 2022). The studies included in the secondary evidence found studied a diverse population in terms of ethnicity and geography. Most of them were observational studies. Moreover, they were characterised by high heterogeneity and different methods of presenting the analysed data were used. It should also be noted that the reviewed publications were created in relation to the cultural and economic context, as well as according to the way the local health care system functions.

## 6. Conclusions

CRC is a severe problem both for the individual and for the general population. Colorectal cancer, through its classic symptoms, significantly reduces the patient’s quality of life. At the same time, CRC generates high direct and indirect costs related to the treatment and medical leave of patients, burdening the public budget and increasing social costs. Therefore, the introduction of appropriate systemic solutions aimed at the early detection of CRC (including FIT screening) allows for the implementation of effective treatment and reduction of the consequences of the disease.

FIT is an effective and cost-effective method of conducting population-wide CRC screening. It is an alternative or complementary to other screening tests, including colonoscopy.

FIT screening should be performed in the asymptomatic population aged 50–75 years, preferably at two-year intervals. The optimal cut-off value for the FIT test is 10 µg/g. If the result indicates CRC, a colonoscopy should be performed no later than 6 months after receiving a positive FIT result.

## Figures and Tables

**Table 1:** Characteristics and results of tests in regard to the diagnostic precision of FIT in the detection of CRC.

Author/Year	Number and Type of Studies	Population			FIT Cut-Off (µg/g)	FIT-Brand	Result	
		Description		Population Size			Sensitivity% (95% CI)	Specificity% (95% CI)
Lin 2021 [8] (MA)	13 observational studies	Asymptomatic people aged ≥ 40 years have a general risk of developing CRC		44,887	-	OC-Sensor	74 (64–83)	94 (93–96)
	4 observational studies			32,424		OC-Light	81 (70–91)	93 (91–96)
Meklin 2020 [12] (MA)	24 observational studies	General		87,073	-	-	86 (78–93)	85 (81–88)
Niedermaier 2020 [13] (MA)	10 cohort studies	People at moderate risk of CRC (population screening) who underwent colonoscopy		203	-	-	I/A: 75 (56–88)	87 (75–94)
							II/B: 77 (63–87)	
							III/C: 85 (65–94)	
							IV/D: 79 (42–95)	
	17 cohort studies	Symptomatic patients who underwent colonoscopy		799	-	-	I/A: 79 (68–86)	87 (83–90)
							II/B: 88 (80–93)	
							III/C: 85 (75–91)	
							IV/D: 87 (76–93)	
	11 case-control studies	Patients diagnosed with CRC who underwent colonoscopy		1228	-	-	I/A: 64 (50–76)	89 (85–92)
							II/B: 80 (74–84)	
							III/C: 82 (77–87)	
							IV/D: 79 (70–86)	
	27 cohort studies, 11 case-control studies	All of the above patients (Total)		2230	-	-	I/A: 73 (65–79)	89 (85–92)
							II/B: 80 (74–84)	
							III/C: 82 (77–87)	
							IV/D: 79 (70–86)	
Imperiale 2019 [15] (MA)	10 observational studies	Asymptomatic people at moderate risk of CRC, at the age of screening (usually between 50 and 75 years of age) who have participated in colonoscopy screening		8364	<10	-	78 (63–88)	90 (81–95)
	16 observational studies			50,892	10	-	91 (84–95)	90 (86–93)
	7 observational studies			12,727	>10–<20	-	82 (63–92)	93 (91–95)
	14 observational studies			56,638	20	-	75 (61–86)	95 (92–96)
	12 observational studies			17,341	>20	-	71 (56–83)	95 (94–96)
	7 observational studies			6715	≤10	OC-Sensor	86 (75–93)	90 (86–93)
	4 observational studies			3890	>10–<20		81 (55–94)	93 (91–93)
	11 observational studies			27,827	20		77 (66–85)	94 (91–96)
	7 observational studies			4347	>20		73 (48–89)	95 (94–96)
	5 observational studies			3428	10	OC-Light	90 (72–97)	91 (83–95)
	1 observational study			4260	≤10	OC-Hemodia	89 (72–96)	94 (93–95)
	1 observational study			3090	>10–<20		53 (32–73)	87 (86–89)
	1 observational study			3794	20		25 (6–57)	96 (96–97)
	2 observational studies			4260	>20		70 (47–86)	97 (96–97)
Selby 2019 [16] (MA)	18 observational studies	Asymptomatic adults screened for CRC		447 *	≤10	-	80 (76–83)	91 (89–93)
	26 observational studies			432 *	>10–≤20	-	69 (63–75)	94 (93–96)
	12 observational studies			188 *	>10–≤30	-	73 (62–81)	96 (95–97)
	8 observational studies			188 *	>30	-	66 (55–75)	96 (94–97)
	8 observational studies			14,407	≤10	OC-Sensor /OC-Micro	31 (25–38)	92 (88–95)
	13 observational studies			49,510	>10–≤20		71 (64–78)	94 (92–96)
	3 observational studies			5029	>20		64 (26–90)	96 (95–97)
	3 observational studies			4267	-	FOB Gold	95 (60–100)	90 (85–94)
	3 observational studies			30,301	-	Magstream	91 (31–100)	94 (92–95)
	6 observational studies			67,894	-	OC-Hemodia	68 (47–83)	96 (93–98)
	3 observational studies	Asymptomatic adults screened for CRC	Women	1,459,185	-	-	81 (60–100)	94 (91–97)
			Men	1,459,185	-	-	77 (75–79)	92 (89–95)
	3 observational studies		At the age of 50–59	1,393,499	-	-	85 (71–99)	94 (92–97)
			At the age of 60–69	1,393,499	-	-	73 (71–75)	93 (90–96)
	46 observational studies	All of the above patients screened for CRC (Total)		2,412,518	-	-	76 (72–80)	94 (92–95)
Stonestreet 2019 [17] (MA)	8 observational studies	Adults with symptoms of gastrointestinal disease and asymptomatic adults over 50 years of age		34,186	-	-	69 (54–81)	94 (94–95)
Katsoula 2017 [18] (MA)	1 RCT, 11 observational studies	Asymptomatic patients with a family history of CRC or a history of polypectomy		4872	-	-	93 (53–99)	91 (59–99)

* number of colorectal cancer cases—stratified by positivity threshold, limited to cohorts with colonoscopy follow-up. MA—meta-analysis; CI—confidence interval; RCT—randomised controlled trial; FIT—faecal immunochemical test; I–IV/AD—stage of colorectal cancer.

**Table 2:** Characteristics and results of studies on reporting for screening and detection of CRC.

Author/Year	N Studies	Population		Screening Method		End Point	OR/RR Score (95% CI) **
		Description	Size (n/N) *	Intervention	Comparator		
Mutneja 2021a [9] (MA)	5 RCT	Patients > 50 years of age	65,368/122,264 (I); 47,025/114,498 (C)	FIT	FS	Reportability for screening	**OR = 2.11 (1.29–3.44)**
			266/65,368 (I); 255/47,025 (C) per protocol			CRC detection indicator	**OR = 0.76 (0.61–0.96)**
			266/122,264 (I); 254/114,498 (C) intention-to-treat				OR = 1.15 (0.65–2.02)
Zhong 2020 [14] (MA)	6 RCT	People at medium risk of CRC (aged 59–69)	19,233/46,189 (I); 8081/36,853 (C)	FIT	Colonoscopy	Reportability for screening	**RR = 1.73 (1.29–2.34)**
			57/19,169 (I); 52/8043 (C) per protocol			CRC detection indicator	**RR = 0.53 (0.33–0.83)**
			54/45,955 (I); 55/36,639 (C) intention-to-treat				RR = 0.73 (0.37–1.42)

* n = case; N = number of people in the intervention or control group ** results in bold indicate statistical significance. MA—meta-analysis; SR—systematic review; CI—confidence interval; RCT—randomised controlled trial; RR—risk ratio; FIT—faecal immunochemical test; FS—flexible sigmoidoscopy; CRC—colorectal cancer; (I)—group examined; (C)—group control.

**Table 3:** Characteristics and test results in regards to death due to CRC.

Author/Year	Number of Studies	Population		Screening Method	End Point	RR Score (95% CI) *
		Characteristics of the Population	Population Size			
Lin 2021 [8] (MA)	cohort study	Asymptomatic people aged ≥40 years have a general risk of developing CRC	5,417,699	FIT	Death due to CRC	RR = 0.90 (0.84–0.95)
Gini 2020 [11] (SR)	1 RCT	People invited to or participating in CRC screening	10,283	FS + FIT		RR = 0.75 (0.57–0.99)
Zhang 2017 [19] (MA)	1 case-control study, 2 cohort studies	General population with an average risk of developing CRC	5,460,619	FIT		RR = 0.41 (0.29–0.59)
	2 cohort studies		75,396	FIT	Incidence of CRC	RR = 0.79 (0.69–0.92)

* Results in bold indicate statistical significance. CI—confidence interval; FIT—faecal immunochemical test; FS—flexible sigmoidoscopy; MA—meta-analysis; SR—systematic review; RCT—randomised controlled trial; RR—risk ratio; CRC—colorectal cancer.

## Data Availability

Not applicable.

## Supplementary Materials

The following supporting information can be downloaded at: https://www.mdpi.com/article/10.3390/cancers14184391/s1, Table S1: AMSTAR 2 rating.
