# Supplementary material for: Clinical Effectiveness of Faecal Immunochemical Test in the Early Detection of Colorectal Cancer—An Umbrella Review
Source: Cancers (Basel). 2022 Sep 9;14(18):4391. doi: 10.3390/cancers14184391 (PMC9496929; doi:10.3390/cancers14184391)
Supplement: Supplementary file 1 [file cancers-14-04391-s001.zip › cancers-1871414-supplementary.pdf]

Search strategy Cochrane

ID	Keyword	Result
#1	MeSH descriptor: [Colonic Neoplasms] explode all trees	1863
#2	MeSH descriptor: [Adenomatous Polyps] explode all trees	259
#3	MeSH descriptor: [Colorectal Neoplasms] explode all trees	9008
#4	#1 OR #2 OR #3	9057
#5	("colon cancer" OR "colorectal cancer" OR "adenomatous polyp*" OR "hereditary non-polyposis colorectal cancer" OR "familial adenomatous polyposis" OR "colonic neoplasia" OR "colorectal carcinoma"):ti,ab,kw	17592
#6	#4 OR #5	20553
#7	("fecal immunochemicy test"):ti,ab,kw	385
#8	(FIT):ti,ab,kw	28016
#9	#7 OR #8	28071
#10	MeSH descriptor: [Mass Screening] explode all trees	4058
#11	("screen*"):ti,ab,kw	83129
#12	#10 OR #11	83471
#13	#6 AND #9 AND #12	487
#14	#6 AND #9 AND #12 in Cochrane Reviews, Publication Date 28.02.2012-28.02.2022	2

Search strategy Medline (via PubMed)

ID	Keyword	Result
#1	Search: Colonic Neoplasms[MeSH Terms]	79244
#2	Search: Adenomatous Polyps[MeSH Terms]	8463
#3	Search: Colorectal Neoplasms[MeSH Terms]	222256
#4	Search: ((Colonic Neoplasms[MeSH Terms]) OR (Adenomatous Polyps[MeSH Terms])) OR (Colorectal Neoplasms[MeSH Terms])	222891
#5	Search: "colon cancer"[Title/Abstract] OR "colorectal cancer"[Title/Abstract] OR "adenomatous polyp*"[Title/Abstract] OR "hereditary non-polyposis colorectal cancer"[Title/Abstract] OR "familial adenomatous polyposis"[Title/Abstract] OR "colonic neoplasia"[Title/Abstract] OR "colorectal carcinoma"[Title/Abstract]	172125
#6	Search: (((Colonic Neoplasms[MeSH Terms]) OR (Adenomatous Polyps[MeSH Terms])) OR (Colorectal Neoplasms[MeSH Terms])) OR ("colon cancer"[Title/Abstract] OR "colorectal cancer"[Title/Abstract] OR "adenomatous polyp*"[Title/Abstract] OR "hereditary non-polyposis colorectal cancer"[Title/Abstract] OR "familial adenomatous polyposis"[Title/Abstract] OR "colonic neoplasia"[Title/Abstract] OR "colorectal carcinoma"[Title/Abstract])	275122
#7	Search: "fecal immunochemical test"[Title/Abstract]	785
#8	Search: FIT[Title/Abstract]	142712

#9	Search: ("fecal immunochemical test"[Title/Abstract]) OR (FIT[Title/Abstract])	142894
#10	Search: Mass Screening[MeSH Terms]	139041
#11	Search: "screen*"[Title/Abstract]	871234
#12	Search: (Mass Screening[MeSH Terms]) OR ("screen*"[Title/Abstract])	913583
#13	Search: (((((Colonic Neoplasms[MeSH Terms]) OR (Adenomatous Polyps[MeSH Terms])) OR (Colorectal Neoplasms[MeSH Terms])) OR ("colon cancer"[Title/Abstract] OR "colorectal cancer"[Title/Abstract] OR "adenomatous polyp*"[Title/Abstract] OR "hereditary non-polyposis colorectal cancer"[Title/Abstract] OR "familial adenomatous polyposis"[Title/Abstract] OR "colonic neoplasia"[Title/Abstract] OR "colorectal carcinoma"[Title/Abstract])) AND (("fecal immunochemical test"[Title/Abstract]) OR (FIT[Title/Abstract]))) AND ((Mass Screening[MeSH Terms]) OR ("screen*"[Title/Abstract]))	1388
#14	Search: (((((((systematic[Title]) AND ((Review[Title/Abstract]) OR "Review" [Publication Type])))))))	187642
#15	Search: ((((((((metaanalysis[Title/Abstract]) OR Meta-Analysis[Title/Abstract]) OR "Meta-Analysis" [Publication Type]))))))	227965
#16	Search: ((((((((systematic[Title]) AND ((Review[Title/Abstract]) OR "Review" [Publication Type])))))))) OR (((((((((metaanalysis[Title/Abstract]) OR Meta-Analysis[Title/Abstract]) OR "Meta-Analysis" [Publication Type])))))))	326020
#17	Search: ((((((Colonic Neoplasms[MeSH Terms]) OR (Adenomatous Polyps[MeSH Terms])) OR (Colorectal Neoplasms[MeSH Terms])) OR ("colon cancer"[Title/Abstract] OR "colorectal cancer"[Title/Abstract] OR "adenomatous polyp*"[Title/Abstract] OR "hereditary non-polyposis colorectal cancer"[Title/Abstract] OR "familial adenomatous polyposis"[Title/Abstract] OR "colonic neoplasia"[Title/Abstract] OR "colorectal carcinoma"[Title/Abstract])) AND (("fecal immunochemical test"[Title/Abstract]) OR (FIT[Title/Abstract]))) AND ((Mass Screening[MeSH Terms]) OR ("screen*"[Title/Abstract]))) AND (((((((((systematic[Title]) AND ((Review[Title/Abstract]) OR "Review" [Publication Type])))))))) OR (((((((((metaanalysis[Title/Abstract]) OR Meta-Analysis[Title/Abstract]) OR "Meta-Analysis" [Publication Type]))))))))	67
#18	Search: ((((((Colonic Neoplasms[MeSH Terms]) OR (Adenomatous Polyps[MeSH Terms])) OR (Colorectal Neoplasms[MeSH Terms])) OR ("colon cancer"[Title/Abstract] OR "colorectal cancer"[Title/Abstract] OR "adenomatous polyp*"[Title/Abstract] OR "hereditary non-polyposis colorectal cancer"[Title/Abstract] OR "familial adenomatous polyposis"[Title/Abstract] OR "colonic neoplasia"[Title/Abstract] OR "colorectal carcinoma"[Title/Abstract])) AND (("fecal immunochemical test"[Title/Abstract]) OR (FIT[Title/Abstract]))) AND ((Mass Screening[MeSH Terms]) OR ("screen*"[Title/Abstract]))) AND (((((((((systematic[Title]) AND ((Review[Title/Abstract]) OR "Review" [Publication Type])))))))) OR (((((((((metaanalysis[Title/Abstract]) OR	63

	Meta-Analysis[Title/Abstract]) OR "Meta-Analysis" [Publication Type])))))))) Filters: from 2012/2/28 - 2022/2/28	

Search strategy Embase (via Ovid)

ID	Keyword	Result
1	exp colon tumor/	343776
2	exp adenomatous polyp/	9513
3	1 or 2	347303
4	("colon cancer" or "colorectal cancer" or "adenomatous polyp*" or "hereditary non-polyposis colorectal cancer" or "familial adenomatous polyposis" or "colonic neoplasia" or "colorectal carcinoma").ab,kw,ti.	249244
5	3 or 4	378472
6	"fecal immunochemical test".ab,kw,ti.	1370
7	FIT.ab,kw,ti.	171650
8	exp occult blood test/	6209
9	6 or 7 or 8	176706
10	exp mass screening/	265913
11	"screen* ".ab,kw,ti.	1170297
12	10 or 11	1273707
13	5 and 9 and 12	5379
14	limit 13 to ((meta analysis or "systematic review") and yr="2012 - 2022")	176

List of studies included and excluded after full-text analysis

Lp.	Authors, Title, Journal	Full text status
1	Katsoula, A. (2017). Diagnostic accuracy of fecal immunochemical test in patients at increased risk for colorectal cancer ameta-Analysis. JAMA Internal Medicine, 177, 1110-1118.	Included
2	Zhang, J. (2017). Effectiveness of Screening Modalities in Colorectal Cancer: A Network Meta-Analysis. Clinical Colorectal Cancer, 16, 252-263.	Included
3	Meklin, J. (2020). Fecal occult blood tests in colorectal cancer screening: Systematic review and meta-analysis of traditional and new-generation fecal immunochemical tests. Anticancer Research, 40, 3591-3604.	Included
4	Gini, A., Jansen, E. E. L., Zielonke, N., Meester, R. G. S., Senore, C., Anttila, A. et al. (2020). Impact of colorectal cancer screening on cancer-specific mortality in Europe: A systematic review. Eur.J.Cancer, 127, 224-235.	Included
5	Imperiale, T. F., Gruber, R. N., Stump, T. E., Emmett, T. W., & Monahan, P. O. (2019). Performance Characteristics of Fecal Immunochemical Tests for Colorectal Cancer and Advanced Adenomatous Polyps: A Systematic Review and Meta-analysis. Ann.Intern.Med., 170, 319-329.	Included
6	Lin, J. S. (2021). Screening for Colorectal Cancer: Updated Evidence Report and Systematic Review for the US Preventive Services Task Force. JAMA - Journal of the American Medical Association, 325, 1978-1997.	Included
7	Niedermaier, T. (2020). Stage-Specific Sensitivity of Fecal Immunochemical Tests for Detecting Colorectal Cancer: Systematic Review and Meta-Analysis. American Journal of Gastroenterology, 115, 56-69.	Included
8	Stonestreet, J. (2019). Systematic review and meta-analysis: Diagnostic accuracy of faecal immuno-chemical testing for haemoglobin (FIT) in detecting colorectal cancer for both symptomatic and screening population. Acta Gastro-Enterologica Belgica, 82, 291-299.	Included
9	Mutneja, H. R. (2021). Comparative effectiveness of fecal immunochemical tests versus flexible sigmoidoscopy for colorectal cancer screening: A systematic review and meta-analysis of randomized clinical trials. Journal of Gastrointestinal and Liver Diseases, 30, no.	Included

**10**	**Mutneja H. R., Bhurwal A., Arora S. et al. (2021). A delay in colonoscopy after positive fecal tests leads to higher incidence of colorectal cancer: A systematic review and meta-analysis. J. Gastroenterol. Hepatol. 36(6): 1479-1486**	**Included**
**11**	**Zhong G. C., Sun W. P., W. L. et al. (2020). Efficacy and cost-effectiveness of fecal immunochemical test versus colonoscopy in colorectal cancer screening: a systematic review and meta-analysis. Gastrointest. Endosc. 91(3): 684-697**	**Included**
**12**	**Selby K., Levine E. H., Doan C. et al. (2019). Effect of Sex, Age and Positivity Threshold on Fecal Immunochemical Test Accuracy: a Systematic Review and Meta-Analysis. Gastroenterology. 157(6): 1494-1505**	**Included**
**13**	**Forbes, N. & Heitman, S. J. (2021). Association Between Time to Colonoscopy After Positive Fecal Testing and Colorectal Cancer Outcomes: A Systematic Review. Clinical Gastroenterology and Hepatology, 19, 1344-1354.**	**Included**
14	Iannone, A. (2016). Stool Investigations for Colorectal Cancer Screening: From Occult Blood Test to DNA Analysis. Journal of Gastrointestinal Cancer, 47, 143-151.	Excluded
15	Gies, A. & Gies, A. (2018). Quantitative fecal immunochemical tests for colorectal cancer screening. International Journal of Cancer, 143, 234-244.	Excluded
16	Pickhardt, P. J. (2021). PPV and detection rate of mt-sDNA testing, FIT, and CT colonography for advanced neoplasia: A hierarchic Bayesian meta-analysis of the noninvasive colorectal screening tests. American Journal of Roentgenology, 217, 817-830.	Excluded
17	Vieito, N. P. (2019). High-risk symptoms and quantitative faecal immunochemical test accuracy: Systematic review and meta-analysis. World Journal of Gastroenterology, 25, 2383-2401.	Excluded
18	Niedermaier, T. (2018). Fecal immunochemical tests in combination with blood tests for colorectal cancer and advanced adenoma detection-systematic review. United European Gastroenterology Journal, 6, 13-21.	Excluded
19	Niedermaier, T. (2016). Fecal Immunochemical Tests Combined with Other Stool Tests for Colorectal Cancer and Advanced Adenoma Detection: A Systematic Review. Clinical and Translational Gastroenterology, 7, no.	Excluded

20	Rabeneck, L. (2012). Fecal immunochemical tests compared with guaiac fecal occult blood tests for population-based colorectal cancer screening. Canadian Journal of Gastroenterology, 26, 131-147.	Excluded
21	GuimarĂŁes, D. P., Fregnani, J. H., Reis, R. M., Taveira, L. N., Scapulatempo-Neto, C., Matsushita, M. et al. (2019). Comparison of a New-generation Fecal Immunochemical Test (FIT) With Guaiac Fecal Occult Blood Test (gFOBT) in Detecting Colorectal Neoplasia Among Colonoscopy-referral Patients. Anticancer Res., 39, 261-269.	Excluded
22	Jodal, H. C. (2019). Colorectal cancer screening with faecal testing, sigmoidoscopy or colonoscopy: A systematic review and network meta-analysis. BMJ Open, 9, no.	Excluded
23	Lee, J. K. (2014). Accuracy of fecal immunochemical tests for colorectal cancer systematic review and meta-Analysis. Annals of Internal Medicine, 160, 171-181.	Excluded
24	Vasilyev, S., Smirnova, E., Popov, D., Semenov, A., Eklund, C., Hendolin, P. et al. (2015). A New-Generation Fecal Immunochemical Test (FIT) Is Superior to Quaiac-based Test in Detecting Colorectal Neoplasia Among Colonoscopy Referral Patients. Anticancer Res., 35, 2873-2880.	Excluded

AMSTAR2

The systematic reviews included in the analysis received the following ratings:

- high – Zhong 2020, Gini 2020, Selby 2019;
- low – Lin 2021, Forbes 2021, Meklin 2020, Imperiale 2019, Katsoula 2017;
- critically low – Mutneja 2021a, Mutneja 2021b, Niedermaier 2020, Stonestreet 2019, Zhang 2017.

Table S1. AMSTAR 2 rating

Publication	Item 2	Item 4	Item 7	Item 9	Item 11	Item 13	Item 15	Overall rating
Lin 2021 (MA)	Yes	Yes	No	Yes	Yes	Yes	Yes	**Low**
Mutneja 2021a (MA)	No	Partial Yes	No	Yes	Yes	Yes	Yes	**Critically Low**
Mutneja 2021b (MA)	No	Partial Yes	No	Yes	Yes	Yes	Yes	**Critically Low**
Forbes 2021 (SR)	Partial Yes	Partial Yes	No	Yes	-	Yes	-	**Low**
Gini 2020 (SR)	Yes	Yes	Yes	Yes	-	Yes	-	**High**

Publication	Item 2	Item 4	Item 7	Item 9	Item 11	Item 13	Item 15	Overall rating
Niedermaier 2020 (MA)	No	Yes	No	Yes	Yes	Yes	Yes	**Critically Low**
Zhong 2020 (MA)	Partial Yes	Yes	Yes	Yes	Yes	Yes	Yes	**High**
Meklin 2020 (MA)	Partial Yes	Partial Yes	No	Yes	Yes	Yes	Yes	**Low**
Imperiale 2019 (MA)	Partial Yes	Partial Yes	No	Yes	Yes	Yes	Yes	**Low**
Selby 2019 (MA)	Yes	Yes	Partial Yes	Yes	Yes	Yes	Yes	**High**
Stonestreet 2019 (MA)	No	No	Partial Yes	Yes	No	Yes	Yes	**Critically Low**
Katsoula 2017 (MA)	Yes	Partial Yes	No	Yes	Yes	Yes	Yes	**Low**
Zhang 2017 (MA)	No	Partial Yes	No	Yes	Yes	Yes	Yes	**Critically Low**

MA – meta-analysis; SR – systematic review

Critical domains: item 2 – protocol registered before commencement of the review; item 4 – adequacy of the literaturę search; item 7 – justyfication for excluding individual studies; item 9 – risk of bias from individual studies being included in the review; item 11 – appropriateness of meta-analytical methods; item 13 – consideration of risk of bias when interpreting the results of the review; item 15 – assessment of presence and likely impact of publication bias.
